# No More Insomnia

**Published:** 1889-12-07

**Authors:** 


### THERAPEUTICS.

### NO MORE INSOMNIA!

Scarcely a month passes without some new hypnotic being brought into—we say deliberately—the market, each with a flourish of trumpets proclaiming its superiority to all previously in use, and its alleged freedom from the drawbacks incident to those that have established their reputation. Unfortunately, they are widely employed, thanks to the advertisements of the wholesale druggists, before they have been submitted to the tests of physiological experiment, or of clinical observation carefully conducted on a sufficient number of individuals under like conditions; and the testimonials appended to the advertisements are mostly based on a few isolated experiences. Dr. Knoblauch, resident physician in the Psychiatric Clinique of the University of Heidelberg, has done good service by publishing his observations on one of the more generally known—viz., sulphonal, introduced about three years ago by Baumann and Kast, and already the subject of no fewer than sixty "reports" in the medical press. The chief objections to sulphonal are, according to Dr. Knoblauch, the long time that elapses before the hypnotic effects appear, the prolongation of the sleep when at length obtained beyond the physiological needs of the organism, the patients sleeping till late in the forenoon, and the feeling of lassitude and weariness that persists for the remainder of the day. Kast ascribed this delay to the insolubility of the drug in cold water, and advised its administration early in the evening in a quantity of hot soup or milk. Ruscheweyh, however, found no acceleration of its action follow the observance of these directions, though smaller doses produced better results than larger ones given in cold water. Even this advantage was not observed by Knoblauch.

The physiological action of sulphonal on animals had been investigated by Shick, in America, and by Dautheville, in France, but more recently and fully by Funaioli and Raimondi, in Italy. They found that like doses produced very dissimilar effects, not only on different animals, but on the same individual at different times, an uncertainty that was fully confirmed by Knoblauch's observations on man. Doses, however, of 0·1—0·2 grams for guinea-pigs, of 0·51—·0 grams for rabbits, and of 2·0—3·0 grams for medium-sized dogs, never failed to induce the characteristic symptoms of weakness and want of co-ordination of the muscles of the hind legs and loins, extending, perhaps, to the fore limbs. This paresis was well marked long before the approach of drowsiness; indeed, the animals were sometimes unnaturally lively and excited—a dog thus manifestly half paralysed caused much amusement by hunting a rabbit in like condition round the garden without apparent fatigue. But soon their eyelids dropped, they staggered as if drunk, and laid down to sleep; the ataxy persisting for some hours after they awoke. Larger doses caused paralysis, sleep, or coma, tremors of the extremities coming on with laboured respiration, then chronic spasm of the jaws. The progress of recovery was the same whatever the dose, but when death ensued it was preceded by a period during which the spasms became weaker and less frequent, till they ceased altogether. The primary action of the drug was certainly on the spinal cord. Similar symptoms have been observed in man by Bornemann, Fischer, and Rehm. Knoblauch has seen them, not indeed after single large doses, but after the continued use of the drug for several weeks. They were inco-ordination of the legs, weakness of the arms, giddiness, grinding of the teeth, and difficulty in speaking. On one occasion they appeared suddenly after two months' daily employment of small doses, and in another, four days after the last of about a dozen doses spread over a month. He used it in twenty cases, including ten of melancholia, three of mania, three of organic psychoses, and four of other forms of insanity. In eight, including the three of mania, two of melancholia, and the three organic cases, no good whatever was done; indeed, the hallucinations in the three last named were greatly intensified. In the remainder sleep was obtained, but the results were certainly not so satisfactory as those following the use of chloral or morphia. In melancholia opium especially had proved beneficial in its effects on the mental state of his patients, while full doses of alcohol Knoblauch has found of great service in moderating hallucinations. Even the absence of ill effects on the heart is unproven, clinical physicians differing in their opinions as to its action in compensatory and non-compensatory forms of heart disease repectively. Dr. Knoblauch is convinced that, owing to the slowness of its action and its uncertainty, it will soon fall into oblivion, and that it is in every respect inferior to chloral and morphine.

Other observations on the physiological action of chloralamid and chloral-urethan, compounds which break up in water, are most unsatisfactory. The former causes extreme dilatation of the arterioles and capillaries with but a short sleep, and experiments with the latter on dogs showed a fall in the blood pressure within an hour from 105 to 50 mm. of mercury, though the sleep obtained was of short duration.

